# Intraplantar aminoglutethimide, a P450scc inhibitor, reduced the induction of mechanical allodynia in a rat model of thrombus-induced ischemic pain

**DOI:** 10.1186/s13041-024-01125-2

**Published:** 2024-08-02

**Authors:** Soon-Gu Kwon, Hoon-Seong Choi, Seo-Yeon Yoon, Dae-Hyun Roh, Jang-Hern Lee

**Affiliations:** 1 Neuracle Science Co., Ltd, Seoul, 02841 Korea; 2 Research Animal Resource Center, Korea Institute of Science and Technology, Seoul, 02792 Korea; 3 Department of Companion Animal Health, Yuhan University, Bucheon-si, 14780 Gyeonggi-do Korea; 4 Department of Oral Physiology, School of Dentistry, Kyung Hee University, Seoul, 02447 Korea; 5 Department of Veterinary Physiology, BK21 PLUS Program for Creative Veterinary Science Research, Research Institute for Veterinary Science and College of Veterinary Medicine, Seoul National University, Seoul, 08826 Korea

**Keywords:** Ischemic pain, Neuroactive steroid, Aminoglutethimide, Cytochrome P450 side-chain cleavage, Sigma-1 receptor

## Abstract

Neuroactive steroids (NASs) directly affect neuronal excitability. Despite their role in the nervous system is intimately linked to pain control, knowledge is currently limited. This study investigates the peripheral involvement of NASs in chronic ischemic pain by targeting the cytochrome P450 side-chain cleavage enzyme (P450scc). Using a rat model of hind limb thrombus-induced ischemic pain (TIIP), we observed an increase in P450scc expression in the ischemic hind paw skin. Inhibiting P450scc with intraplantar aminoglutethimide (AMG) administration from post-operative day 0 to 3 significantly reduced the development of mechanical allodynia. However, AMG administration from post-operative day 3 to 6 did not affect established mechanical allodynia. In addition, we explored the role of the peripheral sigma-1 receptor (Sig-1R) by co-administering PRE-084 (PRE), a Sig-1R agonist, with AMG. PRE reversed the analgesic effects of AMG during the induction phase. These findings indicate that inhibiting steroidogenesis with AMG alleviates peripheral ischemic pain during the induction phase via Sig-1Rs.

**Supplementary Information:**

The online version contains supplementary material available at 10.1186/s13041-024-01125-2.

## Main text

Neuroactive steroids (NASs) are a class of steroids that can modify neuronal excitability in both the central and peripheral nervous systems [1, 2]. The initial rate-limiting step in NAS biosynthesis is regulated by the cytochrome P450 cholesterol side-chain cleavage enzyme (P450scc), which catalyzes the conversion of cholesterol to pregnenolone (PREG). Both NASs and P450scc are involved in the normal functioning of central nervous systems as well as in various pathological conditions, including chronic neuropathic pain induced by peripheral nerve injury [3]. However, the role of P450scc in the peripheral nervous system, where the initial cause of pain originates, remains underexplored.

Peripheral ischemia is common causes of pain in the lower extremities [4]. Previously, we developed a peripheral ischemic pain model named the “thrombus-induced ischemic pain (TIIP) model,” which mimicked the pathogenesis of peripheral ischemic pain in humans [5]. Using this model, we focused on the pain mechanism in context of peripheral sensitization and examined related receptors, such as acid-sensing ion channels, ATP receptors, and sigma-1 receptors (Sig-1Rs), which are known as primary target receptors of NASs [5, 6]. In current study, based on the fact that NASs have been reported to control ischemic damage in the nervous system [7], we hypothesized that intraplantar injection of P450scc inhibitor could modulate ischemic pain at the peripheral site. Therefore, the present study was designed to investigate the potential role of the P450scc in the peripheral ischemic pain and to explore the involvement of Sig-1Rs at the peripheral site.

**Fig. 1:**
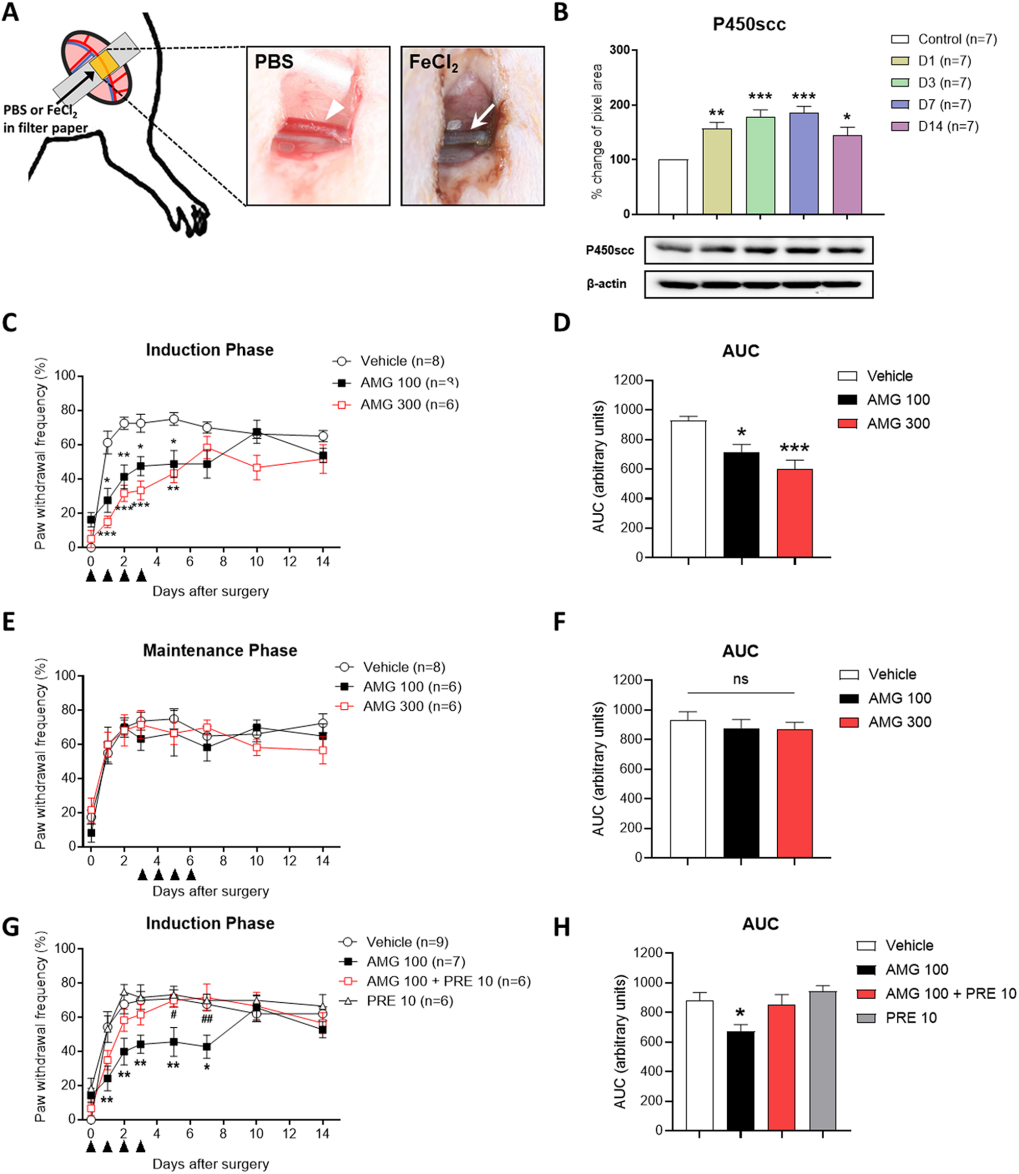
Schematic diagram for surgery of thrombus-induced ischemic pain (TIIP) injury and the effect of intraplantar aminoglutethimide (AMG) and PRE-084 (PRE) on TIIP induced mechanical allodynia. (**A**) Schematic diagram for surgery of thrombus-induced ischemic pain (TIIP) injury. In femoral arteries, normal conditions are observed with PBS treatment (arrowhead), whereas FeCl2-treatment in animals results in the development of thrombus formation or occlusion (arrow). (**B**) Representative Western blots images of P450scc (top) and β-actin (bottom) expression and the graph depicts the P450scc expression levels in the ipsilateral skin on post-operative days 1, 3, 7, and 14. Compared to the sham control group, significant differences were observed from postoperative day 1, and P450scc expression gradually increased until day 7 and then showed decreased trend on day 14. (**C**) During the induction phase, repeated AMG (100 and 300 nmol) treatments inhibited the increase in PWF (%) compared to vehicle-treated TIIP rats. After treatment, the inhibitory effect of AMG on mechanical allodynia gradually recovered. On day 7 after surgery, there was no significant difference between the AMG-treated and vehicle-treated groups. (**D**) The area under curve (AUC) data analysis demonstrated a dose-dependent antiallodynic effect of AMG treated on induction phase of TIIP. (**E**) During the maintenance phase, daily AMG (100 and 300 nmol) treatment did not alleviate the ischemia-induced increase in PWF (%) to mechanical stimuli. (**F**) The AUC data analysis also showed that the treatment of AMG on maintenance phase did not effective on mechanical allodynia in TIIP. (**G**) In TIIP rats, co-administration of AMG (100 nmol) and PRE (10 nmol) reversed AMG’s analgesic effect during the induction phase of mechanical allodynia. A single daily injection of AMG (100 nmol) inhibited the induction of mechanical allodynia in TIIP rats while there was no significant difference between AMG (100 nmol) + PRE (10 nmol) compared to the vehicle-treated group. Repeated injections of PRE (10 nmol) had no significant effect on mechanical allodynia during the induction phase. (**H**) The area under curve (AUC) data analysis showed diminished anti-allodynic effect of AMG (100 nmol) by co-administration of AMG (100 nmol) and PRE (10 nmol) on induction phase of TIIP (*n* = 6 ~ 8 per group). *p < 0.05, and **p < 0.01, and ***p < 0.001 compared to the vehicle-treated group, #*p* < 0.05, and ##*p* < 0.01 compared to the AMG 100 group), All values are expressed as mean ± standard error of the mean (SEM)

Male Sprague–Dawley rats (250–350 g) were anesthetized with 3% isoflurane in a N_2_O/O_2_ gas mixture, and small incision was made in the femoral triangle of the left hind limb. Femoral artery was carefully separated from the vein and nerve, and a filter paper disc (0.5 × 0.5 cm) soaked in a 20% FeCl_2_ in sterile PBS was applied to the femoral artery for 20 min. We defined the time periods in the TIIP model during which mechanical sensitivity increases or is sustained as the ‘induction phase’ (Day 0 to 3) and the ‘maintenance phase’ (Day 4 to 14), respectively (Fig. 1A). 30 µl of either aminoglutethimide (AMG, a P450scc inhibitor) or PRE-084 (PRE, a Sig-1R agonist) were subcutaneously injected daily into the ischemic paw during induction or maintenance phase. A von Frey filament (4.0 g) was applied 10 times to sole of ischemic paw, and results are presented as a percentage of the paw withdrawal frequency (PWF %). Western blot analysis was performed with the ipsilateral skin and temporal expression pattern of P450scc was quantified with Metamorph software (Molecular Devices, Sunnyvale, CA, USA). Statistical analysis was conducted using Prism 9.5 software (GraphPad, San Diego, CA, USA). A one-way ANOVA or two-way ANOVA was used to analyze the data as shown in the figure legend. A *p*-value of less than 0.05 was considered statistically significant.

The temporal expression level of P450scc on the ipsilateral skin exhibited a gradual increase following ischemia. Immunoblot analysis revealed that P450scc levels peaked on post-operative day 7. Although there was still a significant difference compared to the control group, P450scc expression showed a decreasing trend by post-operative day 14 (Fig. 1B). To investigate the role of P450scc in the ischemic pain, pharmacological intervention was applied by administering AMG (100 and 300 nmol) directly into the ischemic hind paw during induction or maintenance phase of TIIP. During the induction phase, repeated AMG treatments significantly inhibited the increase in PWF (%) in TIIP rats (Fig. 1C). The AUC analysis also showed that AMG treatment dose-dependently inhibited the increase in mechanical allodynia (Fig. 1D). The inhibitory effect of AMG on mechanical allodynia gradually diminished after treatment. However, during the maintenance phase, AMG had no effect on the established mechanical allodynia in TIIP rats (Fig. 1E and F).

To investigate the role of peripheral Sig-1Rs in AMG-induced pain inhibition in induction phase, PRE (10 nmol) was co-administered with AMG (100 nmol) during the induction phase. Co-administration of PRE with AMG reversed the analgesic effect observed in the AMG-treated group on post-operative days 5, and 7 (Fig. 1G). Additionally, AUC analysis demonstrated that the combined treatment of PRE and AMG reversed the anti-allodynic effect of AMG in TIIP (Fig. 1H).

Skin is one of the main extra-gonadal steroidogenic organs that contains the P450scc enzyme [8]. Previous studies have shown that P450scc expression in skin can be regulated by external irritants [9]. Our current research further indicates increased P450scc levels following peripheral ischemic insult. It is noteworthy that peripheral nerves express various NASs and enzymes, including P450scc, which function as physiological regulators and protective agents for the peripheral nerve [7]. Consequently, the elevated peripheral P450scc observed in TIIP rats may be part of a physiological mechanism that provides protection against nerve damage resulting from ischemia.

Sig-1Rs are primary target receptors for NAS such as PREG and dehydroepiandrosterone (DHEA) [10]. We previously reported that peripherally administered PREG sulfate (PREGS) and DHEA sulfate (DHEAS) enhance pain sensitivity and it is closely related with peripheral Sig-1Rs activation [11]. Thus, it is plausible that PREGS and DHEAS contribute to mechanical allodynia during the induction phase of TIIP. Intraplantar administrated AMG inhibits the activation of P450scc, consequently reducing peripheral PREGS and DHEAS levels, which results in a decrease in mechanical allodynia by attenuating peripheral Sig-1R activity. Regarding the role of Sig-1R in TIIP, we previously demonstrated that inhibition of peripheral Sig-1Rs alleviated the mechanical allodynia only during the induction phase of TIIP [6]. Moreover, peripheral Sig-1Rs expression was increased peaking on day 3 and returning to baseline levels by day 7 [6]. These findings suggest that peripheral Sig-1Rs play a limited role in the induction phases of TIIP. Therefore, considering the role of Sig-1R in ischemic pain, which is specific to the induction phase, despite a significant increase in the expression of P450scc from day 1 to 14, pharmacological inhibition of P450scc could only impact the initial induction phase of TIIP.

Overall, our study demonstrated that peripheral P450scc level was increased in ischemic damage and intraplantar injection of AMG significantly alleviated mechanical allodynia during the induction of TIIP through Sig-1Rs activation. These findings indicate that peripheral NASs plays a pivotal role in peripheral sensitization and could represent a novel therapeutic target for chronic ischemic pain.

### Electronic supplementary material

Below is the link to the electronic supplementary material.

Supplementary Material 1

Supplementary Material 2

Supplementary Material 3

## Data Availability

All data supporting the findings are included and are available on request from the corresponding author.

## References

[1] Baulieu EE, Neurosteroids. A novel function of the brain. Psychoneuroendocrino. 1998;23(8):963–87. 10.1016/S0306-4530(98)00071-7 9924747

[2] Dubrovsky B. Neurosteroids, neuroactive steroids, and symptoms of affective disorders. Pharmacol Biochem Be. 2006;84(4):644–55. 10.1016/j.pbb.2006.06.016 16962651

[3] Choi SR, Beitz AJ, Lee JH. Inhibition of cytochrome P450 side-chain cleavage attenuates the development of mechanical Allodynia by reducing spinal D-Serine production in a Murine Model of Neuropathic Pain. Front Pharmacol. 2019;10. 10.3389/fphar.2019.01439 PMC6908476 31866864

[4] Lang PM, Schober GM, Rolke R, Wagner S, Hilge R, Offenbächer M, et al. Sensory neuropathy and signs of central sensitization in patients with peripheral arterial disease. Pain. 2006;124(1–2):190–200. 16716518 10.1016/j.pain.2006.04.011

[5] Seo HS, Kim HW, Roh DH, Yoon SY, Kwon YB, Han HJ, et al. A new rat model for thrombus-induced ischemic pain (TIIP); development of bilateral mechanical allodynia. Pain. 2008;139(3):520–32. 18691814 10.1016/j.pain.2008.06.011

[6] Kwon SG, Roh DH, Yoon SY, Choi SR, Choi HS, Moon JY, et al. Role of peripheral sigma-1 receptors in ischaemic pain: potential interactions with ASIC and P2X receptors. Eur J Pain. 2016;20(4):594–606. 26358747 10.1002/ejp.774

[7] Borowicz KK, Piskorska B, Banach M, Czuczwar SJ. Neuroprotective actions of neurosteroids. Front Endocrinol (Lausanne). 2011;2:50. 22649375 10.3389/fendo.2011.00050 PMC3355955

[8] Slominski AT, Manna PR, Tuckey RC. On the role of skin in the regulation of local and systemic steroidogenic activities. Steroids. 2015;103:72–88. 25988614 10.1016/j.steroids.2015.04.006 PMC4631694

[9] Shimada-Omori R, Yamasaki K, Koike S, Yamauchi T, Aiba S. TLR3 augments glucocorticoid-synthetic enzymes expression in epidermal keratinocytes; implications of glucocorticoid metabolism in rosacea epidermis. J Dermatol Sci. 2020;100(1):58–66. 32888783 10.1016/j.jdermsci.2020.08.011

[10] Maurice T. Neurosteroids and σ receptors, biochemical and behavioral relevance. Pharmacopsychiatry. 2004;37:S171–82. 15547783 10.1055/s-2004-832675

[11] Kwon SG, Yoon SY, Roh DH, Choi SR, Choi HS, Moon JY, et al. Peripheral neurosteroids enhance P2X receptor-induced mechanical allodynia via a sigma-1 receptor-mediated mechanism. Brain Res Bull. 2016;121:227–32. 26876754 10.1016/j.brainresbull.2016.02.012

