# Supplementary material for: Intraplantar aminoglutethimide, a P450scc inhibitor, reduced the induction of mechanical allodynia in a rat model of thrombus-induced ischemic pain
Source: Mol Brain. 2024 Aug 2;17:50. doi: 10.1186/s13041-024-01125-2 (PMC11295590; doi:10.1186/s13041-024-01125-2)
Supplement: Supplementary file 1 — Supplementary Material 1 [file 13041_2024_1125_MOESM1_ESM.docx]

**Supplementary Figure**


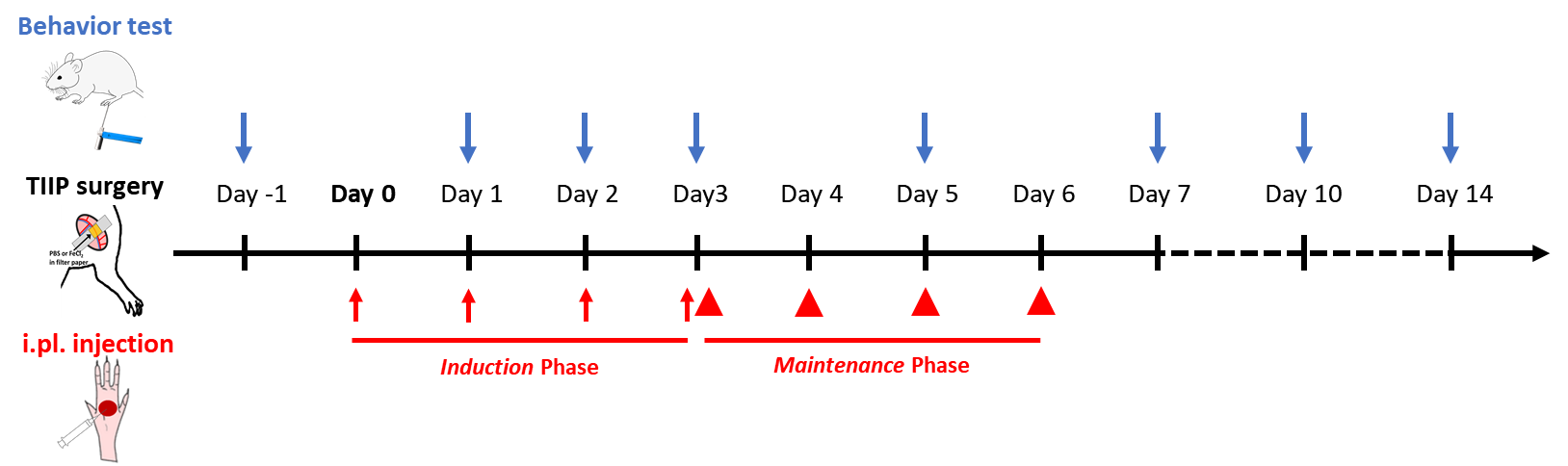


**Fig. S1** The experimental time schedule for behavior test and intraplantar (i.pl.) aminoglutethimide (AMG) and PRE-084 (PRE) injection. Paw withdrawal frequency was determined for all rats before TIIP injury at the day before surgery to obtain normal baseline. Rats were then tested at day 1, 2, 3, 5, 7, 10, and 14 post-TIIP injury. Intraplantar injection of AMG and PRE was performed during the ‘induction phase’ (day 0 to 3) or ‘maintenance phase’ (day 3 to 6), respectively, once a day.
