# Supplementary material for: Intraplantar aminoglutethimide, a P450scc inhibitor, reduced the induction of mechanical allodynia in a rat model of thrombus-induced ischemic pain
Source: Mol Brain. 2024 Aug 2;17:50. doi: 10.1186/s13041-024-01125-2 (PMC11295590; doi:10.1186/s13041-024-01125-2)
Supplement: Supplementary file 2 — Supplementary Material 2 [file 13041_2024_1125_MOESM2_ESM.docx]

**Supplementary Table 1**

**Table 1** Effect of aminoglutethimide (AMG) on induction or maintenance phase of mechanical allodynia in the thrombus-induced ischemic pain


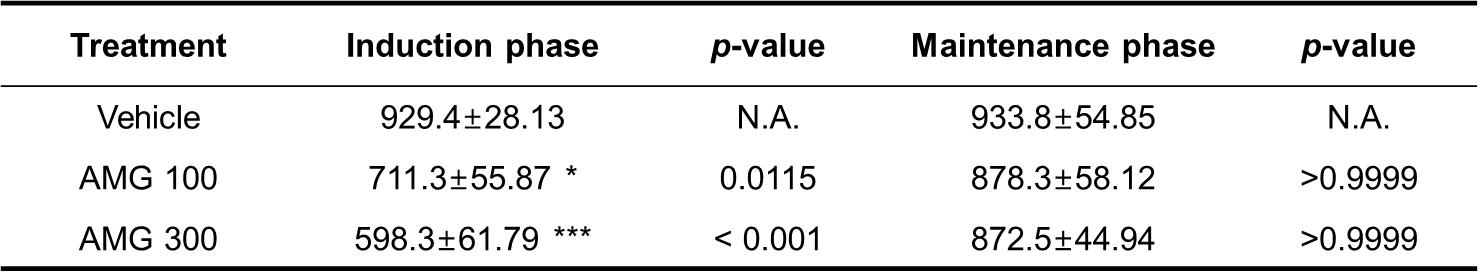


Notes: All values are mean ± S.E.M using one-way ANOVA followed by Bonferroni test; **p* < 0.05 and ****p* < 0.001. Abbreviations: ANOVA, analysis of variance; N.A., not applicable; S.E.M, standard error of the mean
