# Supplementary material for: Intraplantar aminoglutethimide, a P450scc inhibitor, reduced the induction of mechanical allodynia in a rat model of thrombus-induced ischemic pain
Source: Mol Brain. 2024 Aug 2;17:50. doi: 10.1186/s13041-024-01125-2 (PMC11295590; doi:10.1186/s13041-024-01125-2)
Supplement: Supplementary file 3 — Supplementary Material 3 [file 13041_2024_1125_MOESM3_ESM.docx]

**Supplementary Table 2**

**
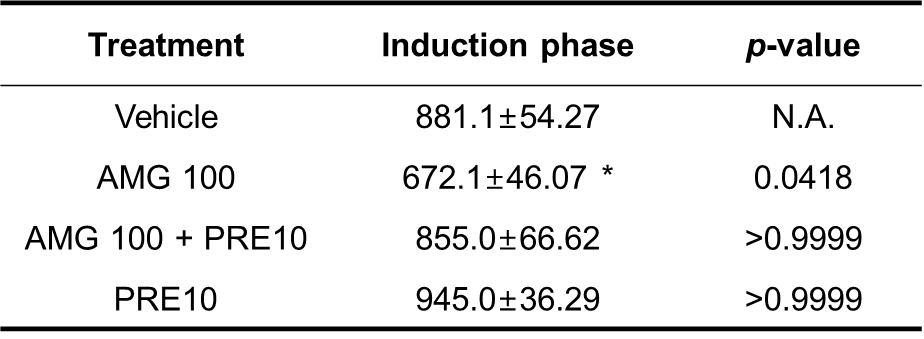
Table 2**. Effect of PRE-084 (PRE) on aminoglutethimide (AMG) mediated inhibitory effect of mechanical allodynia in the induction phase of thrombus-induced ischemic pain

Notes: All values are mean ± S.E.M using one-way ANOVA followed by Bonferroni test; ***p* < 0.01. Abbreviations: ANOVA, analysis of variance; N.A., not applicable; S.E.M, standard error of the mean
